# Prostate Cancer Among Black Men in Canada

**DOI:** 10.1001/jamanetworkopen.2024.18475

**Published:** 2024-06-25

**Authors:** Patrick Albers, Safaa Bashir, Nikhile Mookerji, Stacey Broomfield, Anaïs Medina Martín, Sunita Ghosh, Adam Kinnaird

**Affiliations:** 1 Division of Urology, Department of Surgery, University of Alberta, Edmonton, Alberta, Canada; 2 Black Medical Students Association, Department of Medicine, University of Alberta, Edmonton, Alberta, Canada; 3 Alberta Prostate Cancer Research Initiative, Alberta, Canada; 4 Department of Oncology, University of Alberta, Edmonton, Alberta, Canada; 5 Cancer Research Institute of Northern Alberta, Edmonton, Alberta, Canada; 6 Alberta Centre for Urologic Research and Excellence, Edmonton, Alberta, Canada

## Abstract

**Importance:**

Prostate cancer is a prevalent disease among men worldwide, exhibiting substantial heterogeneity in presentation and outcomes influenced by various factors, including race and ethnicity. Disparities in incidence, stage at diagnosis, and survival rates have been observed between Black men and those of other races and ethnicities.

**Objective:**

To compare prostate cancer outcomes between Black men and men with other race (Asian, Hispanic, Indigenous, Middle Eastern, White, Multiracial, and Other) in a universal health care system, with race and ethnicity self-reported.

**Design, Setting, and Participants:**

This was a prospective, observational cohort study of men diagnosed with prostate cancer between June 1, 2014, and August 28, 2023, who self-identified race and ethnicity. Participants included men who had been prospectively enrolled in the Alberta Prostate Cancer Research Initiative from the 2 major urology referral centers in Alberta (University of Alberta and University of Calgary). All men with prostate cancer enrolled in the initiative were included.

**Exposure:**

Race and ethnicity.

**Main Outcomes and Measures:**

The primary outcome was the stage and grade of prostate cancer at diagnosis. Further outcomes included age and prostate-specific antigen level at diagnosis, initial treatment modality, time from diagnosis to initial treatment, and prostate cancer–specific, metastasis-free, and overall survivals.

**Results:**

A total of 6534 men were included; 177 (2.7%) were Black, and 6357 (97.3%) had another race or ethnicity. Men who identified as Black were diagnosed with prostate cancer at an earlier age (mean [SD], 62.0 [8.2] compared with 64.6 [7.7] years; *P* < .001) and had a lower Charlson Comorbidity Index rating (14% compared with 7% ≤ 1; *P* < .001) compared with men of other races. Men who identified as Black had similar prostate-specific antigen levels at diagnosis, TNM category (74% vs 74% with T1-T2; *P* = .83) and Gleason Grade Group (34% compared with 35% Gleason Grade Group 1; *P* = .63). Black men had similar rates of prostate cancer–specific (hazard ratio [HR], 1.10; 95% CI, 0.41-2.97; *P* = .85), metastasis-free (HR, 0.88; 95% CI, 0.42-1.46; *P* = .44), and overall (HR, 0.55; 95% CI, 0.25-1.24; *P* = .15) survival.

**Conclusions and Relevance:**

The findings of this cohort study suggest that Black men, despite being diagnosed at a younger age, experience comparable prostate cancer outcomes compared with men of other races.

## Key Points

**Question:**

Do Black men have worse prostate cancer outcomes compared with men of other races in a universal health care system?

**Findings:**

In this prospective cohort study that included 6534 men with prostate cancer, Black men had prostate cancer outcomes similar to men of other races despite being diagnosed 2.6 years earlier. In a universal health care system, Black men had similar prostate cancer–specific, metastasis-free, and overall survival to men of other races.

**Meaning:**

Despite similar prostate cancer outcomes, Black men are diagnosed with prostate cancer at a younger age and may benefit from earlier prostate cancer screening.

## Teaser

This cohort study compares the stage and grade of prostate cancer at diagnosis among Black men compared with men of other races and ethnicities.

## Introduction

Prostate cancer (PCa) exhibits considerable heterogeneity in its presentation and outcomes, with various factors, such as race and ethnicity, age, comorbidities, and socioeconomic status influencing the progression of the disease and management. Among these factors, race has been a subject of interest due to observed disparities in incidence, stage at diagnosis, and survival rates, with data from the US and Britain showing a double lifetime risk of PCa for Black men compared with White men.^1,2^ The reasons for this disparity are multifaceted, reflecting a complex interplay of socioeconomic, cultural, and inheritable genetic factors.^3,4^ Limited access to quality health care, including screening and early detection programs, is a significant contributor, exacerbated by systemic issues, such as unequal distribution of resources and institutional biases.^5,6,7,8,9^ Additionally, genetic predispositions, hormonal differences, and variations in tumor biology may play a role, although research in this area is ongoing.^10,11,12^ Addressing these disparities requires a comprehensive approach that evaluates both the health care system’s structural inequities and the multifactorial nature of prostate cancer risk among Black men.

The purpose of this study was to explore baseline demographic and cancer characteristics as well as survival outcomes in patients with PCa, focusing on the comparison between Black men and men of differing ethnicities (self-reported as Asian, Hispanic, Indigenous, Middle Eastern, White, Multiracial, and Other [non-Black]) within the context of a Canadian single-payer, government-funded, universal health care system. We aimed to identify potential disparities and elucidate their implications for PCa management and health care resource allocation.

## Methods

### Patients

Patients (N = 6534) were included from the Alberta Prostate Cancer Research Initiative (APCaRI), which is a prospective cohort that has been collected from the 2 major urology referral centers in Alberta (University of Alberta and University of Calgary). All men diagnosed with PCa assessed by specialists at these 2 sites were eligible for inclusion in APCaRI with written informed consent from participants; participants did not receive financial compensation. All patients with PCa enrolled from July 1, 2014, to August 28, 2023, were included in this study. Patients in this cohort self-identified race and ethnicity and are prospectively followed up at least every 6 months from the time of PCa diagnosis. Description of the APCaRI cohort has previously been published.^13^ The study protocol was approved by the Health Research Ethics Board of Alberta. The Strengthening the Reporting of Observational Studies in Epidemiology (STROBE) reporting guideline was followed for cohort studies.

### Outcomes

The primary outcome of this study was stage and grade of PCa at diagnosis. Secondary outcomes in this study included age at diagnosis, prostate-specific antigen (PSA) level at diagnosis, initial treatment modality, time from diagnosis to initial treatment, as well as PCa-specific, metastasis-free, and overall survival.

### Statistical Analysis

Mean (SD) values are reported for continuous variables, and categorical variables are reported as frequencies (percentage). Analysis of variance and *t* tests were used to compare continuous variables where appropriate, and the χ^2^ test was used to compare categorical variables. Survival analyses for metastasis-free survival, PCa-specific survival, and overall survival were conducted and Kaplan-Meier estimates were calculated. A log-rank test was used to compare the Kaplan-Meier curves. Men were analyzed by self-identified race and ethnicity, with men of other races considered in aggregate owing to small sample sizes. SPSS, version 29 (IBM Corp) was used for all statistical analysis. *P* < .05 was used as the threshold for statistical significance, and all hypothesis tests were based on 2-sided tests.

## Results

A total of 6534 men were included; 177 (2.7%) were Black, and 6357 (97.3%) had another race or ethnicity. Patient demographic and tumor characteristics at the time of PCa diagnosis are reported in the Table. In this cohort, Black men were diagnosed with PCa 2.6 years earlier (mean [SD] age, 62.0 [8.2] compared with 64.6 [7.7] years; *P* < .001) and had a lower Charlson Comorbidity Index rating (14% compared with 7% ≤ 1; *P* < .001). There was no significant difference in family history of PCa (31% compared with 33%; *P* = .20), body mass index (calculated as weight in kilograms divided by height in meters squared) (27.8 [IQR, 25.2-31.3] compared with 27.8 [IQR, 25.1-31.2]; *P* = .96), PSA level at diagnosis, TNM category (74% with 74% T1-T2; *P* = .83), Gleason grade group (34% with 35% in Gleason grade group 1; *P* = .63), initial treatment choice, or time from diagnosis to treatment (11 with 10.5 weeks; *P* = .62). There was a shorter duration (approximately 2 months) of follow-up for Black men in this study (median, 39.4 [IQR, 26.4-62.5] with 41.3 [IQR, 27.1-74.4] months; *P* = .02).

**Table.** Baseline Demographic Characteristics

Characteristic	Black men (n = 177)	Men of other races (n = 6357)	*P* value
Age, mean (SD)	62.0 (8.2)	64.6 (7.7)	<.001
BMI, median (IQR)	27.8 (25.2-31.3)	27.8 (25.1-31.2)	.96
Charlson Comorbidity Index score, No (%)			
0	6 (3)	39 (1)	<.001
1	19 (11)	428 (7)	
≥2	152 (86)	5890 (93)	
Race and ethnicity, No. (%)^a^			
Asian	0	394 (6)	<.001
Black	177 (100)	0	
Indigenous	0	86 (1)	
Hispanic	0	48 (1)	
Middle Eastern	0	13	
Multiracial	0	9	
White	0	5786 (91)	
Other/unknown	0	21	
Family history of prostate cancer, No. (%)	54 (31)	2233 (35)	.20
PSA, No. (%), ng/mL			
<10	139 (79)	5201 (82)	.10
10-20	21 (12)	789 (12)	
>20	17 (10)	367 (6)	
Clinical stage, No. (%)			
Tx	13 (7)	363 (6)	.83
T1	87 (49)	3113 (49)	
T2	62 (35)	2200 (35)	
T3/4	9 (5)	384 (6)	
TanyN1	1 (1)	93 (1)	
TanyNanyM1	5 (3)	204 (3)	
Gleason grade group, No. (%)			
1	58 (34)	2178 (35)	.63
2	71 (41)	2467 (39)	
3	29 (17)	901 (14)	
4	5 (3)	267 (4)	
5	9 (5)	454 (7)	
Time from diagnosis to treatment, median (IQR), wk	11 (4-21.8)	10.5 (4-18)	.62
Initial treatment choice, No. (%)			
Active surveillance	35 (24)	1173 (21)	.27
Radical prostatectomy	55 (37)	2033 (36)	
Radiation	48 (32)	1945 (35)	
Primary ADT	10 (7)	256 (5)	
Cryoablation	1 (1)	182 (3)	
Follow-up duration, median (IQR), mo	39.4 (26.4-62.5)	41.3 (27.1-74.4)	.02

Abbreviations: ADT, androgen deprivation therapy; BMI, body mass index (calculated as weight in kilograms divided by height in meters squared); PSA, prostate-specific antigen.

SI conversion factor: To convert PSA to micrograms per liter, multiply by 1.

^a^ Race and ethnicity were self-reported.

**Figure 1.**
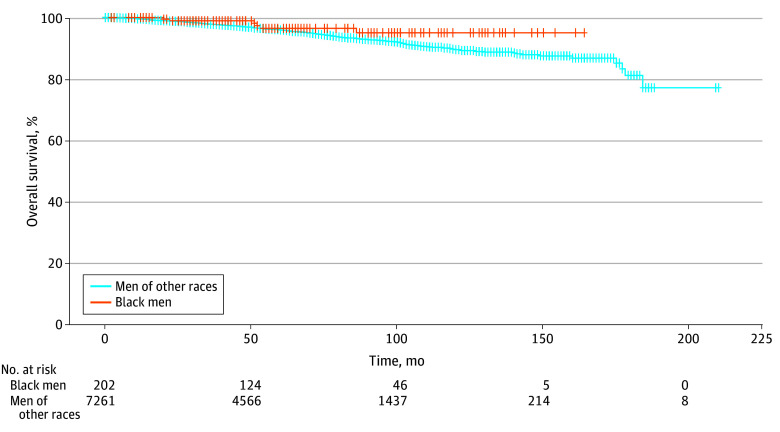
Overall Survival Stratified by Black Men Compared With Men of Other Races Other races comprised Asian, Hispanic, Indigenous, Middle Eastern, White, Multiracial, and Other.

**Figure 2.**
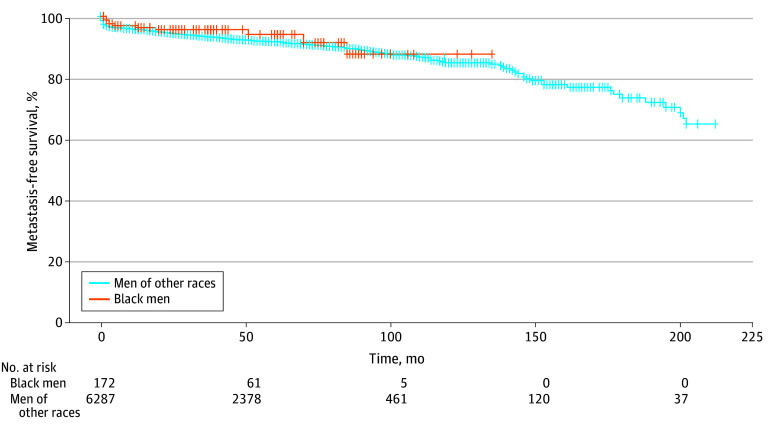
Prostate Cancer Metastasis-Free Survival Stratified by Black Men Compared With Men of Other Races Other races comprised Asian, Hispanic, Indigenous, Middle Eastern, White, Multiracial, and Other.

**Figure 3.**
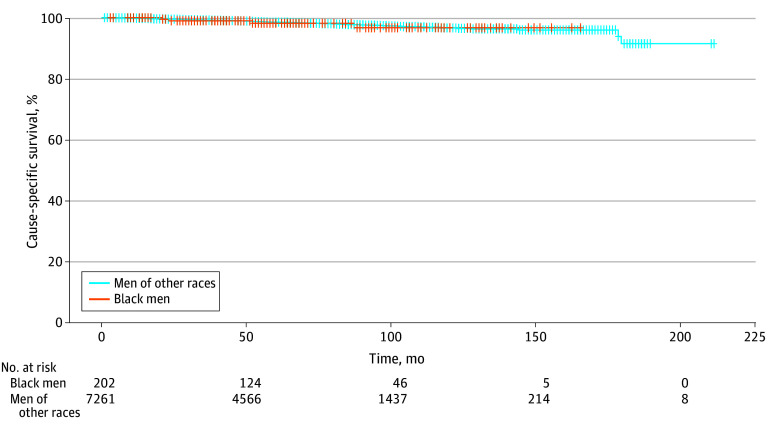
Prostate Cancer–Specific Survival Stratified by Black Men Compared With Non-Black Men Other races comprised Asian, Hispanic, Indigenous, Middle Eastern, White, Multiracial, and Other.

Black men had similar rates of overall survival compared with men of other races in this study (Figure 1) (HR, 0.55; 95% CI, 0.25-1.24; *P* = .15). Black men had similar rates of metastasis-free survival (Figure 2) (HR, 0.88; 95% CI, 0.42-1.46; *P* = .44) and PCa-specific survival (Figure 3) (HR, 1.10; 95% CI, 0.41-2.97; *P* = .85). Multivariate modeling was performed for metastasis-free, overall, and PCa-specific survival (eTable 1, eTable 2, and eTable 3 in Supplement 1). Black men had similar rates of overall survival (HR, 0.70; 95% CI, 0.31-1.59; *P* = .40), metastasis-free survival (HR, 0.79; 95% CI, 0.42-1.47; *P* = .46), and PCa-specific survival (HR, 1.37; 95% CI, 0.51-3.70; *P* = .53).

## Discussion

This study observed equivalent PCa outcomes for Black men compared with men of other races in a Canadian cohort. Black men were diagnosed with PCa at an earlier age and had fewer comorbidities at diagnosis. At diagnosis, Black men had similar family history of prostate cancer, BMI, PSA level at diagnosis, TNM stage, and Gleason grade group at diagnosis. Furthermore, Black men had similar rates of overall, metastasis-free, and PCa-specific survival compared with men of other races. Ideally, a universal health care system would minimize barriers to health care, although another study using the APCaRI cohort found worse PCa-specific outcomes in Indigenous men compared with men of other races and ethnicities.^14^

There have been a variety of studies that have looked at PCa outcomes in Black men and found that they are disproportionately affected by PCa, with earlier presentation, more aggressive disease, and higher mortality rates.^3,15^ There are several hypotheses for these disparities, including biological differences; racial bias; differences in screening, treatment, and access to care; and decreased participation in clinical trials, with the reality likely involving all of these.^3,4^

Guidelines and research have indicated that there might be biological differences that account for some of these disparities.^10,11,12^ A study from England, another country with a universal health care system, found that Black men had an 8% lifetime risk of dying by PCa compared with 4% for White men and only 2% for Asian men.^2^ Another study from England reported that Black men had the highest PCa incidence, at 24.7% compared with 19.8% for White men, although with a similar incidence of PCa diagnosed at an advanced stage.^16^ While a universal health care system cannot account for all confounders, one would anticipate that observed differences may be more likely related to biological factors than socioeconomic factors. This increased lifetime risk of PCa mortality was also seen using data from the American Cancer Society. Black men had an 18.2% lifetime risk of developing PCa and 4.4% lifetime risk of PCa mortality compared with 13.3% of PCa development and 2.4% mortality for non-Hispanic White men.^17,18^

Globally, despite differing rates of PCa screening and health care availability, Black men have significantly higher rates of PCa than men of other races, and PCa is a leading cause of cancer-specific mortality.^19,20,21^ It has also been reported that, even when diagnosed with low-risk PCa, Black men have more aggressive disease characteristics and are more likely to have the cancer upgraded than a man of a different race or ethnicity with low-risk PCa.^22^ This was further highlighted by a study reporting that Black men had significantly higher rates of PCa detection on prostate biopsy with similar PSA levels (49% compared with 39% for non-Hispanic White men at a PSA level of 4.0 ng/mL [to convert to micrograms per liter, multiply by 1]).^23^ Evidence has suggested that Black men with metastatic, castrate-resistant PCa have improved survival with systemic agent treatment compared with White men.^24,25^ Despite this, Black men were less likely to receive treatment compared with White men when the PCa worsened, which is certain to play a part in the disparity of outcomes.^26^

Important to note in this study is the age of PCa diagnosis of Black men is significantly younger than non-Black men. This difference in age at diagnosis could provide evidence for differences in genetic predisposition, with Black men in this cohort being diagnosed, on average, 2.6 years earlier than men of other races. Similar to our results, a large-scale retrospective study using the Surveillance, Epidemiology, and End Results database found Black men were diagnosed with PCa 2 years earlier than men of other races.^27^ Despite no specific recommendation for Black men in the most recent Canadian Urologic Association Guidelines for PCa screening, the European Association of Urology and American Urology Association guidelines recommend an earlier age of screening by 5 years compared with the general screening population.^10,11,12^ While this is not implicit in the Canadian Urologic Association guidelines, many Canadian practice patterns mimic US and European guidelines and could be partially responsible for the younger age at diagnosis for Black men in this cohort.

Genetics in PCa is complex and multifactorial and involves a complex interplay of single nucleotide variants, loci, and allele substitutions that put men at higher risk for PCa.^28,29^ Black men have been found to have higher rates of several of the loci associated with PCa compared with men of other races.^28,30^ A study looking at genetic mutations associated with PCa therapeutic targets found similar rates of mutations between men with African ancestry and European ancestry and the investigators concluded that precision medicine approaches would be beneficial to all patients, regardless of race or ethnicity.^31^ Although there have been some genetic differences observed, there is an underrepresentation of Black men in these studies, which makes drawing conclusions difficult.^32^ Other studies also suggested that men with European ancestry are overrepresented in cancer trials studying genetics, with a review published in 2019 finding that only 37% of these studies reported race and of those that reported race, only 14% of the participants identified as Black.^33^ It is imperative to conduct more extensive association studies within Black Canadian and African American populations to identify risk loci specific to PCa in this demographic cohort.

While biological factors may play a part in these differences, the social determinants of health are certain to play a major role. In the Western world, systemic racism and racial bias have long been barriers to equitable access to health care.^5,6,7,8,9^ A legacy of historical and contemporary mistreatment has led to Black men expressing a heightened level of general skepticism toward the health care system.^34,35^ Studies looking at income and employment have found that in Canada, racialized groups earn less and have higher rates of unemployment compared with nonracialized groups.^36,37^ This in turn will affect overall PCa outcomes, as it has been reported that men with lower socioeconomic status have worse PCa outcomes.^38,39^ A US study observed that, when adjusted for socioeconomic factors and access to health care, Black men had similar rates of PCa-specific mortality.^40^ Their result is similar to this present study and may indicate that socioeconomic factors play a larger role than genetic differences in the disparities of PCa outcomes than initially believed.

A Canadian study highlighted the paucity of PCa data on Black men in the Canadian health care system and an associated overreliance on data from other countries, most commonly US data.^41^ This has been partially rectified with a retrospective publication looking at men from the 1991 Canadian long-form census with data out to 2010.^42^ Black men had similar rates of PCa-specific mortality, and in fact the HR was less than 1 (HR, 0.83; 95% CI, 0.67-1.02). The results from this present study are similar, suggesting any racial differences on the outcomes of PCa in Black Canadians may not be as strong as observed in other health regions.

### Limitations

There are several limitations to this study, including the voluntary nature of enrollment into the APCaRI database, which has led to underrepresentation of Black men (2.7% in this study compared with 4.3% of the Albertan population). This self-selection bias could further result in differences in patient outcomes that may not be accounted for in this study. These data also do not consider areas of the social determinants of health that are known to affect health-related outcomes, such as socioeconomic status, language, and religion, among others.

## Conclusions

In this cohort study, we observed that in a universal health care system, despite being diagnosed at a younger age, Black men had comparable PCa outcomes with men of other races, with similar stage and grade of cancer at diagnosis. The younger age of diagnosis for Black men underlies the benefit of risk adapted screening strategies in men at risk of prostate cancer.

## Notes

Supplement 1. **eTable 1.** Multivariate Analysis of Overall Survival **eTable 2.** Multivariate Analysis of Metastasis-Free Survival **eTable 3.** Multivariate Analysis of Prostate Cancer–Specific Survival

Supplement 2.
Data Sharing Statement
