# Supplementary material for: Prostate Cancer Among Black Men in Canada
Source: JAMA Netw Open. 2024 Jun 25;7(6):e2418475. doi: 10.1001/jamanetworkopen.2024.18475 (PMC11200144; doi:10.1001/jamanetworkopen.2024.18475)
Supplement: Supplement 1. — eTable 1. Multivariate Analysis of Overall Survival eTable 2. Multivariate Analysis of Metastasis-Free Survival eTable 3. Multivariate Analysis of Prostate Cancer–Specific Survival [file jamanetwopen-e2418475-s001.pdf]

Supplementary Online Content

Albers P, Bashir S, Mookerji N, et al. Prostate cancer among Black men in Canada. *JAMA Netw Open*. 2024;7(6):e2418475. doi:10.1001/jamanetworkopen.2024.18475

eTable 1. Multivariate Analysis of Overall Survival

eTable 2. Multivariate Analysis of Metastasis-Free Survival

eTable 3. Multivariate Analysis of Prostate Cancer–Specific Survival

This supplementary material has been provided by the authors to give readers additional information about their work.

© 2024 Albers P et al. *JAMA Network Open.*

eTable 1- Multivariate analysis of Overall survival (Abbreviations: HR, Hazard Ratio)

Variables	HR	95% CI	P-value
**Black vs. men of other races**	0.70	0.31 – 1.59	0.40
**Age**	1.09	1.08 - 1.11	**<0.001**
**PSA**	1.001	1.001 - 1.001	**<0.001**
**Family History**	1.21	0.96 - 1.52	0.11

© 2024 Albers P et al. *JAMA Network Open.*

eTable 2 - Multivariate analysis of metastasis-free survival (Abbreviations: HR, Hazard Ratio)

Variables	HR	95% CI	P-value
**Black vs. men of other races**	0.79	0.42-1.47	0.46
**Age**	0.99	0.99-1.10	0.91
**PSA**	1.00	1.00-1.00	0.41
**Family History**	0.83	0.70-0.99	**0.04**

© 2024 Albers P et al. *JAMA Network Open.*

eTable 3 - Multivariate analysis of Prostate cancer-specific survival (Abbreviations: HR, Hazard Ratio)

Variables	HR	95% CI	P-value
**Black vs. men of other races**	1.37	0.51 – 3.70	0.53
**Age**	1.09	1.06 - 1.11	**<0.001**
**PSA**	1.001	1.001 - 1.001	**<0.001**
**Family History**	1.38	0.92 - 2.06	0.12

© 2024 Albers P et al. *JAMA Network Open.*
