# Supplementary material for: Prostate Cancer Among Black Men in Canada
Source: JAMA Netw Open. 2024 Jun 25;7(6):e2418475. doi: 10.1001/jamanetworkopen.2024.18475 (PMC11200144; doi:10.1001/jamanetworkopen.2024.18475)
Supplement: Supplement 2. — Data Sharing Statement [file jamanetwopen-e2418475-s002.pdf]

Data Sharing Statement

Albers. Prostate Cancer Outcomes Among Black Men in Canada. *JAMA Netw Open*.
Published June 25, 2024. doi:10.1001/jamanetworkopen.2024.18475

Data
Data available: Yes
Data types: Deidentified participant data
How to access data: ask@ualberta.ca
When available: With publication

Supporting Documents
Document types: None

Additional Information
Who can access the data: researchers whose proposed use of the data has been approved
Types of analyses: for any purpose
Mechanisms of data availability: With a signed access agreement
